# Evolutionary study of *Yersinia* genomes deciphers emergence of human pathogenic species

**DOI:** 10.1038/srep36116

**Published:** 2016-10-31

**Authors:** Shi Yang Tan, Irene Kit Ping Tan, Mui Fern Tan, Avirup Dutta, Siew Woh Choo

**Affiliations:** 1 Department of Oral and Craniofacial Sciences, Faculty of Dentistry, University of Malaya, 50603 Kuala Lumpur, Malaysia; 2 Genome Informatics Research Laboratory, High Impact Research Building, University of Malaya, 50603 Kuala Lumpur, Malaysia; 3 Institute of Biological Sciences, Faculty of Science, University of Malaya, 50603 Kuala Lumpur, Malaysia

## Abstract

On record, there are 17 species in the *Yersinia* genus, of which three are known to be pathogenic to human. While the chromosomal and pYV (or pCD1) plasmid-borne virulence genes as well as pathogenesis of these three species are well studied, their genomic evolution is poorly understood. Our study aims to predict the key evolutionary events that led to the emergence of pathogenic *Yersinia* species by analyzing gene gain-and-loss, virulence genes, and “Clustered regularly-interspaced short palindromic repeats”. Our results suggest that the most recent ancestor shared by the human pathogenic *Yersinia* was most probably an environmental species that had adapted to the human body. This might have led to ecological specialization that diverged *Yersinia* into ecotypes and distinct lineages based on differential gene gain-and-loss in different niches. Our data also suggest that *Y. pseudotuberculosis* group might be the donor of the *ail* virulence gene to *Y. enterocolitica*. Hence, we postulate that evolution of human pathogenic *Yersinia* might not be totally in parallel, but instead, there were lateral gene transfer events. Furthermore, the presence of virulence genes seems to be important for the positive selection of virulence plasmid. Our studies provide better insights into the evolutionary biology of these bacteria.

*Yersinia* is a genus of Gram-negative bacteria consisting of at least 17 known species^1^. Among these, *Y. pestis*, *Y. pseudotuberculosis* and *Y. enterocolitica* are pathogenic to human, *Y. ruckeri* is pathogenic to salmonids^2^,^3^, while the other *Yersinia* species are apathogenic^3^. Both *Y. pseudotuberculosis* and *Y. enterocolitica* are enteropathogens that cause gastrointestinal infection and are distantly related to each other^2^. *Y. pestis*, which diverged from *Y. pseudotuberculosis* at least 2,000 years ago, can be transmitted by flea into the bloodstream of mammals, causing three pandemics of plague^4^.

The human pathogenic *Yersinia* species carry the virulence plasmid, called pYV in *Y. enterocolitica* or pCD1 in *Y. pseudotuberculosis* and *Y. pestis*, which encodes the Ysc-Yop type three secretion system (T3SS). T3SS allows pathogenic *Yersinia* to escape phagocytosis and takes control of the signaling systems of the host cells^5^. Other known virulence genes in the *Yersinia* species that cause pathogenesis are the chromosome-borne invasin (*inv*), the attachment-invasion gene (*ail*), pH 6 antigen and the virulence plasmid-borne *yad*A^6^. They encode proteins that mediate adhesion and entry into the host cell lining^6^.

While the virulence genes and pathogenesis of human pathogenic *Yersinia* are well studied, the evolution of the genus and emergence of pathogenic species are poorly understood^5^,^6^,^7^,^8^. A previous model proposed that all human pathogenic *Yersinia* descended from a pathogenic *Yersinia*, without regard to apathogenic species^2^. Later, other studies showed incongruence with the previous model, proposing that both *Y. pseudotuberculosis* group (comprising *Y. pseudotuberculosis* and *Y. pestis*) and *Y. enterocolitica* have evolved independently but acquired similar set of virulence genes^9^,^10^.

In view of the contradictory concepts, we further examine the evolution of *Yersinia* to elucidate (1) the role of the most recent ancestor shared by the human pathogenic species before their divergence, and (2) factors that mediate the acquisition of the virulence genes and virulence plasmid to transform into pathogenic species.

## Results

### Properties of *Yersinia* genomes

A total of 15 complete *Yersinia* genomes were used in this study (Supplementary Table 2). Six of them were human pathogenic strains. The size of these genomes ranged from approximately 3.7 Mbp to 4.9 Mbp, while the average GC content was about 47%. All *Yersinia* had seven rRNA operons, except *Y. pestis* CO92 which had six.

### *Yersinia* phylogeny

**Figure 1:**
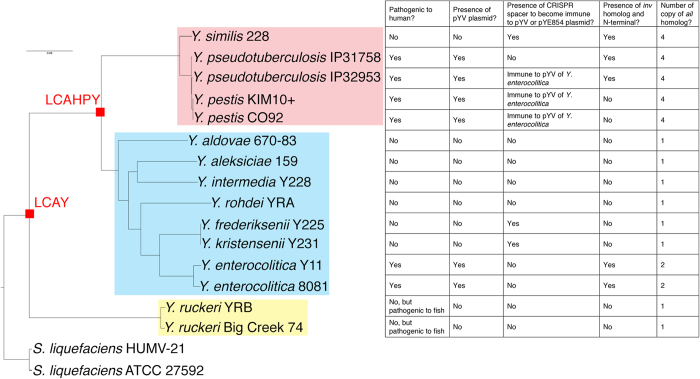
*Yersinia* supermatrix tree rooted using *Serratia liquefaciens* with 100 bootstrap value in every internal node. Phylogroup-P, phylogroup-E, and phylogroup-R are highlighted in magenta, cyan, and yellow respectively. Last Common Ancestor of all *Yersinia* (LCAY) is hypothesized as the most recent hypothetical ancestor shared by all *Yersinia* species while Last Common Ancestor of Human Pathogenic *Yersinia* (LCAHPY) is hypothesized as the most recent hypothetical ancestor shared by human pathogenic *Y. enterocolitica*, *Y. pseudotuberculosis*, and *Y. pestis*. Important properties of each *Yersinia* genome are tabulated on the table to the right of supermatrix tree.

To study the phylogenetic relationship of the 15 *Yersinia* strains, we constructed a phylogenetic tree using a set of concatenated core protein coding sequences with 245,662 nucleotides. Our rooted supermatrix tree clearly showed that the *Yersinia* species could be clustered into three phylogroups that descended from Last Common Ancestor of all *Yersinia* (LCAY): phylogroup-P, phylogroup-E, and phylogroup-R (Fig. 1). Human pathogenic *Y. pseudotuberculosis* group (consisting of *Y. pseudotuberculosis* and *Y. pestis*) and *Y. enterocolitica* belonged to phylogroup-P and phylogroup-E respectively. Besides, the *Y. pseudotuberculosis* group and *Y. enterocolitica* appeared to be at the basal position of the supermatrix tree and closer to the apathogenic species in their respective phylogroups, suggesting that they might have evolved from different apathogenic populations (Fig. 1). We found that the gene content-based phylogenetic tree (Supplementary Fig. 1) had highly similar phyletic patterns with the supermatrix tree (Fig. 1), indicating that different genes are likely borne by the *Yersinia* species of different phylogroups. Thus, we suggest that lateral gene transfer is unlikely to be the major force in shaping the composition of *Yersinia* genomes^11^.

The average relative rate of recombination (R) to mutation (θ) of *Yersinia* genus was estimated to be R/θ = 0.011, mean DNA import length was δ = 603 base pair (bp), mean divergence of imported DNA was ν = 0.041. As R/θ was smaller than 1, mutation is likely a dominant occurrence in the genus, taking place at 90 (1 /0.011 = 90) times more often than recombination. It is possible that recombination across different species would decrease due to the increase of nucleotide divergence between *Yersinia* species^12^.

### Gene gain-and-loss in *Yersinia*

**Figure 2:**
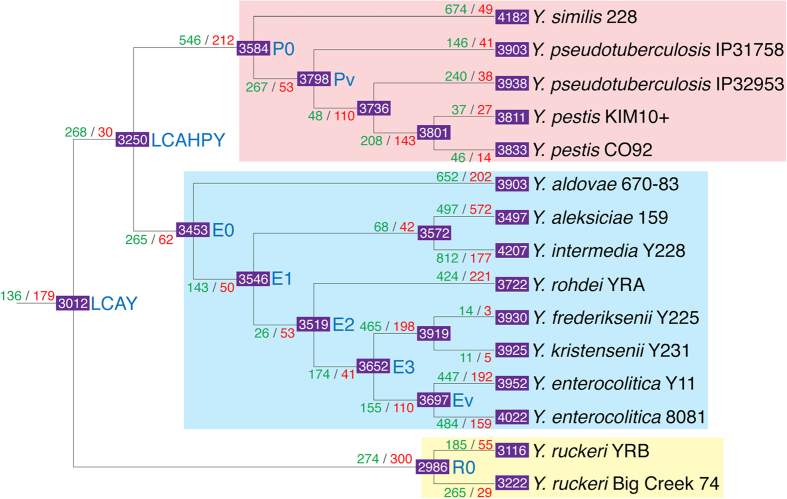
Cladogram shows reconstruction of gene gain-and-loss in *Yersinia*. Green, red, white color numbers indicate gene gain, gene loss and estimated number of gene respectively. Hypothetical ancestors of interest discussed in the main text are labelled in blue color text. Magenta, cyan and yellow backgrounds indicate phylogroup-P, phylogroup-E, and phylogroup-R respectively.

To understand how gene content of *Yersinia* changed since their emergence, we performed gene gain-and-loss analysis to identify acquired and lost genes. Reconstruction of gene gain-and-loss suggests that gene gain was dominant in the evolution of *Yersinia* (Fig. 2). In the next sections, we will discuss the hypothetical ancestors leading to the emergence of pathogenic *Yersinia* in more details.

#### Emergence of LCAY

LCAY is considered the most recent hypothetical ancestor shared by all *Yersinia* species. LCAY might have preferred an aerobic environment due to the acquisition of aerobic citrate transporter genes (*tct*ABCDE)^13^, and might have been able to extract heme from the host organism as indicated by the gain of heme receptor gene (*has*R) and hemophore gene (*has*A)^14^.

Our data showed that LCAY had lost the genes *dsd*AXC which are important for tolerance to D-serine, an anti-microbial compound abundant in the brain and urinary tract, which inhibits growth of enterohemorrhagic *Escherichia coli*^15^. Besides, 3-hydroxy-phenylacetate (3HPA) and 4HPA catabolism genes (*hpa*BCGEDHIAX) were lost, suggesting LCAY no longer used 3HPA and 4HPA in its new niche.

#### Emergence of R0 ancestor

R0 ancestor descended from LCAY and was the first hypothetical ancestor in phylogroup-R. R0 ancestor was found to gain several putative virulence loci including *ysa*-T3SS locus, *yts*1-type two secretion system (T2SS) locus, *ent* locus^16^,^17^. The *ent* locus consists of *ent*ABCES genes which synthesize ruckerbactin and are up regulated when *Y. ruckeri* infects fish^18^. R0 ancestor had also gained genes encoding for anti-sigma factor (*rsb*W) and anti-anti-sigma factor (*rsb*V) that play an important role in osmoprotection of *Streptomyces coelicolor*^19^, probably reflecting the importance of these genes to *Y. ruckeri* since it lives in freshwater.

It should be noted that *Y. ruckeri* has narrower niche as it mainly associates with and infects fishes^3^. This could explain our observation that several metabolic genes and transporters were lost in the R0 ancestor, probably because they were unneeded by *Y. ruckeri* in the more restricted niche. For instance, myo-inositol degradation genes (*iol*ABCDEG) that encode enzymes to degrade myo-inositol, an abundant compound in soil, would probably be no longer useful in freshwater. Another locus, *efe*UOB encoding a ferrous transporter induced under acidic environment, was also lost in the R0 ancestor. This loss might have been due to the shift to freshwater which has more neutral pH^20^.

#### Emergence of LCAHPY

LCAHPY was the last common ancestor shared by human pathogenic *Y. pseudotuberculosis*/*Y. pestis* and *Y. enterocolitica* (Figs 1 and 2). We found that LCAHPY had acquired *pga*ABCD (poly-beta-1,6-N-acetyl-D-glucosamine synthesis and transport genes), *pel* and *pel*W (pectate lyases), *tog*BANM and *tog*T (oligogalacturonide transporter genes). Previous studies have shown that these genes allow human enteric pathogen, such as *Escherichia coli* EDL933, to persist and proliferate on vegetables^21^,^22^,^23^,^24^. Hence, the acquisition of *pga*, *pel* and *tog* loci suggests that LCAHPY may have the capability to grow on vegetables and be introduced into the human gastrointestinal tract after consumption of vegetables.

Besides the above-mentioned genes which facilitated survival outside human intestines, we found LCAHPY ancestor had also acquired *yut* and *urt*ABCDE (urea transporter genes), *yut* and *urt*ABCDE (nickel transporter genes), *ure*ABCEFGD (urease genes). Previous study showed that these genes allowed *Helicobacter pylori* to colonize and cause infections in stomach, suggesting similar role in LCAHPY^25^. The survival of LCAHPY in human gastrointestinal tract could be further enhanced through the acquisition of *lsr*ABCD (autoinducer-2 transporter genes) and *lsr*ABCD (autoinducer-2 processing enzymes genes). Previous study proposed that enteric bacteria may use Lsr proteins to interrupt intercellular communication among competing bacterial cells^26^.

#### Emergence of E0, E1, E2, E3 ancestors

Both phylogroup-E and phylogroup-P descended from LCAHPY. Within phylogroup-E, many hypothetical ancestors (designated E0, E1, E2 and E3 in this study) existed before emergence of human pathogenic *Y. enterocolitica* (Fig. 2). We found that these hypothetical ancestors had acquired *hyb* and *hyf* loci (hydrogenase genes), *cbi*ABCDETFGHJKLMNOQP and *cob*STU (cobalamin biosynthesis genes), *pdu*BCELPQW (1,2-propanediol degradation genes) and *ttr*ABCRS locus (tetrathionate reduction genes). Previous study showed that these loci provided growth advantage to *Salmonella enterica* serotype Typhimurium in the gastrointestinal tract and to outcompete other enteric bacteria^27^. This suggests similar role of these acquired genes during emergence of phylogroup-E species. Besides, our data suggest that cellobiose is important to phylogroup-E as the ancestor had gained second copy of *cel*ABC (cellobiose phosphotransferase system).

#### Emergence of Ev ancestor

Ev was the most recent ancestor shared by human pathogenic *Y. enterocolitica* and it was a descendant of above-mentioned E0, E1 and E3 ancestors in phylogroup-E. Cellobiose seemed to be important to the lifestyle of Ev ancestor because we found Ev ancestor had acquired the third copy of *cel*ABC genes. The pyrimidine catabolic genes (*rut*RABCDEFG) were also acquired by Ev ancestor but their physiological role in bacteria is not yet understood. The absence of *rut* locus in all apathogenic species within phylogroup-E suggests that it might play an important role in the virulence traits of *Y. enterocolitica*. Most importantly, we found that the Ev ancestor had acquired pYV plasmids and several other virulence genes, such as mucoid *Yersinia* factor (Myf) genes and *ail*.

#### Emergence of P0 ancestor

P0 ancestor was the first hypothetical ancestor of phylogroup-P, as well as the direct descendant of LCAHPY. We found P0 ancestor had gained different types of metabolic genes compared to the phylogroup-E species. It gained tellurite resistance genes (*ter*ZABCD) and itaconate catabolic genes (*rip*ABC), which had been shown by previous studies as adaptive strategies to survive inside macrophages^28^,^29^,^30^. Besides, P0 ancestor had gained several virulence genes, including *pil*WVUSRQPONML (type IV pilus gene cluster which resides in *Yersinia* adhesion pathogenicity island), *psa*ABCEF (pH 6 antigen genes). All of these virulence genes had been shown to be important in pathogenicity of human pathogenic *Yersinia*^31^,^32^.

We found P0 had lost *bcs*GFE and *bcs*QABZC which are cellulose synthesis genes. A recent study had demonstrated that repression of cellulose biosynthesis in *Salmonella* when it was inside a macrophage could increase its virulence^33^. It is possible that the loss of cellulose biosynthesis genes and gain of itaconate (antimicrobial compound secreted by macrophage) catabolic genes could enhance survival of phylogroup-P species inside the macrophage.

#### Emergence of Pv ancestor

Pv was the most recent ancestor shared by human pathogenic *Y. pseudotuberculosis* and *Y. pestis* in phylogroup-P. We found that Pv ancestor had acquired *mqs*R and *mqs*A, which are a pair of toxin-antitoxin genes. Previous study has showed that *mqs*R and *mqs*A are the most highly up regulated gene in persistent *E. coli* cells and they regulated other physiological genes^34^. This suggests that the *mqs* toxin-antitoxin gene pair may be important for the pathogenic phylogroup-P species to overcome stresses from the host immune mechanisms.

### Genes exclusive to pathogenic *Yersinia*

We attempted to search for genes exclusive to pathogenic *Yersinia* from different phylogroups. These genes included pYV (or pCD1 in *Y. pseudotuberculosis* and *Y. pestis*) virulence plasmid-borne *yad*A and *ysc*-*yop* T3SS, chromosomal *ybt* locus (yersiniabactin synthesis and transport system genes) and *yts*1-T2SS. Previous studies have demonstrated that both *ybt* and *yts*1-T2SS loci are important to highly human pathogenic *Yersinia*^16^,^17^,^35^.

### *inv* and *ail* homologs within *Yersinia*

**Figure 3:**
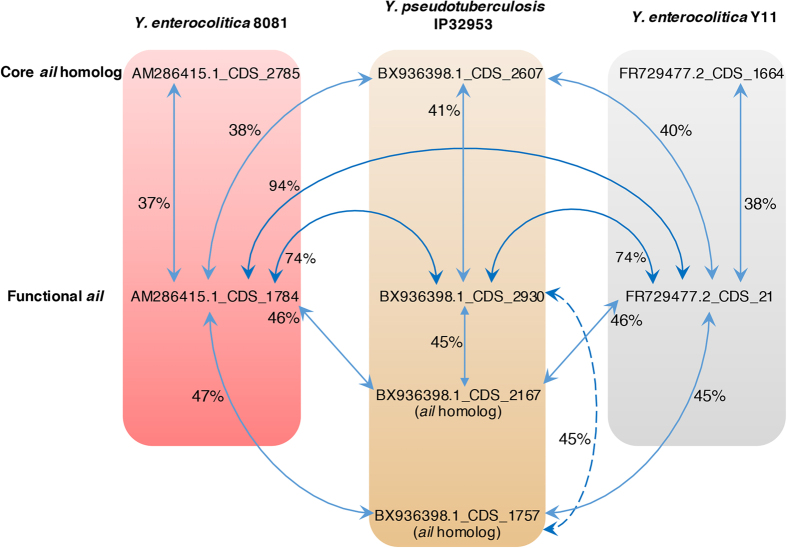
Pairwise percentage of identity between *ail* protein sequences homologs of *Y. pseudotuberculosis* IP32953, *Y. enterocolitica* 8081 and Y11. Pairwise relationships are indicated by blue double arrow pointing to two locus tags while percentage of identity is labeled next to the arrow.

Both Ail and Inv are important virulence factors in human pathogenic *Yersinia* to mediate adhesion and invasion into host cells^6^. Therefore, we attempted to analyze *ail* and *inv* homologs in *Yersinia*. We found a total of 32 genes in *Yersinia* homologous to the functional *ail* from the pathogenic *Y. pestis* CO92, which we used as a reference gene for comparison in this analysis. BLASTP searches of all 32 *ail* homologs against the NCBI NR database showed that *Yersinia* species were always in the top significant hits for each homolog, suggesting that these putative homologs are likely from the *Yersinia* genus^36^. In another BLASTP search for functional *ail* of *Y. enterocolitica* 8081 against all 32 *ail* homologs of *Yersinia*, we found that phylogroup-P species were in the top significant hits (Supplementary Table 4). We further calculated the pairwise sequence identity between all functional *ail* genes and homologs from *Yersinia*. We found that the functional *ail* genes of *Y*. *enterocolitica* Y11 and 8081 were closer to the *ail* and *ail* homologs of the *Y*. *pseudotuberculosis* IP32953 (reference of phylogroup-P species) than their own *ail* homologs (Fig. 3). As the top hits returned by the BLAST program can be used to predict the donor of laterally transferred gene^36^, we thus hypothesize that the *ail* of *Y. enterocolitica* might be originated from the *ail* of *Y. pseudotuberculosis,* for example through lateral gene transfer.

Clustering of the 32 homologs based on protein sequence similarity clearly showed three separate gene clusters (Supplementary Table 3): Cluster 1 consists of known *ail* from both *Y. pestis* and *Y. pseudotuberculosis* and one *ail*-homolog from the *Y. similis*; the Cluster 2 consists of known *ail* from *Y. enterocolitica* and two *ail*-homologs from the *Y. similis*; Cluster 3 consists of the core *ail*-homologs present in all *Yersinia*. In the Cluster 1, we found that all pathogenic *Yersinia* species from the phylogroup-P had their own known *ail* with two copies of *ail*-homologs in each genome, suggesting that these genes are in-paralogs that were likely acquired through duplication events before the divergence of these species from their Pv ancestor.

We found that apathogenic *Yersinia* species generally had *inv* homologs. However, our data showed that there is a difference between *inv* homolog of apathogenic *Yersinia* and the functional *inv* of human pathogenic *Yersinia*. For instance, the aligned region between *inv* homologs of all apathogenic *Yersinia* (except *Y. similis*) and the functional *inv* of the pathogenic species did not start at first amino acid (Supplementary Table 5). We believe that this might account for different expression of the protein transcribed from *inv* homolog in apathogenic species as the N-terminal of Inv is required for proper localization in the outer membrane of *Yersinia*^37^.

### CRISPR-Cas system in *Yersinia*

The CRISPR-Cas system is known to be a defense mechanism for bacteria to become immune to phage and plasmid^38^. Spacers which located in CRISPR array can provide resistance to foreign DNA if there is sequence homology between them. Analyses on the spacers found in *Yersinia* revealed that they could provide immunity against pYE854 conjugative and pYV virulence plasmid (Supplementary Table 6). pYE854 has been demonstrated to be self-transmissible, and is able to mobilize pYV plasmid^39^. The loss of CRISPR-Cas system in *Y. enterocolitica* suggests the event might be one of the key factors to acquire the pYV plasmid. Besides, apathogenic *Y. similis* 228 had spacers that were similar to virulence plasmids of pathogenic *Y. enterocolitica* and *Y. pseudotuberculosis* group. We suggest that these spacers inhibited the acquisition of pYV (or pCD1) plasmids by *Y. similis* after its divergence from P0 ancestor. The spacers in *Y. pseudotuberculosis* IP32953 and *Y. pestis* were found to be similar to the pYV plasmid from *Y. enterocolitica* (Supplementary Table 6), suggesting the phylogroup-P species could fragment and fail to maintain the pYV plasmid from *Y. enterocolitica* if the plasmid is transferred laterally.

### Analyses of pYV (or pCD1) virulence plasmid

We further analyzed the pYV (or pCD1 in *Y. pseudotuberculosis* and *Y. pestis*) virulence plasmids because they are the key to pathogenesis in *Yersinia* species. The virulence plasmids encode for the hostile Ysc-Yop T3SS that takes over the host cell signaling system^5^. We found that the virulence plasmids borne by both phylogroup-P and phylogroup-E species had very high pairwise average nucleotide identity (ANI) values (e.g. >95%) and highly similar GC contents, suggesting that these virulence plasmids are likely closely related even though they were borne by different human pathogenic *Yersinia* species^40^ (Supplementary Tables 7 and 8).

The clustering of protein sequences encoded by these virulence plasmids showed that 53 out of a total of 128 gene families (<50%) were core genes or conserved across all species. The number of accessory genes and strain-specific genes in the plasmid gene families were 30 and 45 respectively, suggesting that these genes are not conserved across all pathogenic *Yersinia* species. The core genes mainly encoded for Ysc-Yop T3SS, whereas several transposase and integrase genes were found in the accessory gene pool. Among the strain-specific genes in the pYV plasmid, we found arsenic detoxification genes (*ars*CBRH) borne by *Y. enterocolitica* Y11, which is consistent with a previous study suggesting that the presence of the *ars* locus might be important for the spread of low pathogenic *Y. enterocolitica*^41^.

## Discussions

Ecological speciation has been proposed to be a major mechanism in explaining diversification of prokaryotes, and lateral gene transfer is recognized as an important force to acquire niche-specific genes, yielding nascent populations in new niches^42^. In this study, the highly similar topology between the supermatrix tree (Fig. 1) and gene content phylogenetic tree (Supplementary Fig. 2) suggests that the lateral gene transfer between phylogroups might not be extensive^11^.

Our data suggest that LCAHPY, the most recent ancestor of human pathogenic *Yersinia*, had adapted to live in the human gastrointestinal tracts. This could be an important milestone in the evolution of *Yersinia* since the environment can provide a wide range of nutrients and niches to the bacterial populations, allowing subpopulations to exploit different food in new niches relative to the ancestral one. As a result, different metabolic genes have been gained and lost in the P0 and E0-3 ancestors throughout the evolution time^42^.

We found that the phylogroup-P species seemed to have expanded their ecological niche to macrophages, probably due to the acquisition of putative genes such as *ter* and *rip* loci, and the loss of two cellulose biosynthesis loci (*bcs*), which could also increase virulence inside the macrophages^28^,^29^,^30^,^33^. This might allow the phylogroup-P species to occupy the macrophage compared to its predecessor and phylogroup-E, which usually adapt to the intestinal tracts. This adaptation could be another efficient way to divide resources for utilization between the two different phylogroups and add weight to the ecological speciation.

During the ecological speciation process, the genetic recombination and gene flow between bacterial populations of different niches might still be possible, preventing them to diverge into distinct lineages^42^. However, in our estimation of the rate of recombination analysis, we clearly showed that the mutations play a major role in causing elevated nucleotide divergence in these *Yersinia* phylogroups. Therefore, this could be a barrier for sexual mating between these *Yersinia* species^12^.

Differentiation of sub-populations in response to new ecological niches may not fully explain nor justify the transformation into pathogenic species. Our analysis suggests that the loss of the CRISPR-Cas system might be critical in mediating the acquisition of the pYV (or pCD1) virulence plasmid in *Yersinia*. However, this might also introduce two enigmatic questions: (1) Most *Yersinia* species have lost the CRISPR-Cas system, but why do they have no virulence plasmids? (2) Some apathogenic *Yersinia* species had CRISPR-Cas system and spacer, but the spacer might be mutated and could decrease the efficiency of their CRISPR-Cas system. Will this allow the bacteria to acquire the virulence plasmid? Answers to these questions pertain to the redundancy of virulence plasmids in the human apathogenic *Yersinia*. For instance, *Y. rukeri* is known to be only pathogenic to salmonids and does not have the pYV plasmid. However, genes in the pYV (or pCD1) plasmids are usually induced at 37 °C^5^, but the salmon bodies do not reach such high temperature. In this case, the pYV is unlikely to be beneficial to and could be redundant or costly for the *Y. ruckeri* to bear it. Moreover, the pYV (or pCD1)-encoded Ysc-Yop T3SS proteins require direct physical contact between the bacteria and host cells for the effector proteins to be injected, and also require several virulence loci to assist in delivery of Yop proteins^5^,^6^. We found that none of the human apathogenic *Yersinia* had functional virulence genes, e.g. *inv* and *ail*. For example, although human apathogenic *Yersinia* species have *inv* homologs, but they are nonfunctional. It could be due to lack of proper N-terminal at the beginning of its protein product. Therefore, the apathogenic *Yersinia* are unlikely to be able to adhere to and invade the cell lining of the host if they accidentally acquire the virulence plasmids. If the physical contact and invasion are not established, the acquisition of virulence plasmid would be redundant for the human apathogenic species. In summary, we believe that the loss or mutation of CRISPR-Cas system might increase the chance of the acquisition of pYV (or pCD1) virulence plasmid by *Yersinia* species. However, to maintain the virulence plasmid, it must first be favored for selection because it is costly for bacteria to bear plasmid^43^. Thus, the presence of the important functional virulence genes, as well as the ability of Ysc-Yop T3SS to express at 37 °C environment could also become important factors determining the successful acquisition of pYV (or pCD1) virulence plasmid.

Our data support the view that gene duplication may play important evolutionary role in the *ail* of human pathogenic *Yersinia*. The *ail* genes in the pathogenic phylogroup-P species might have been aroused from gene duplication. Multiple copies of such *ail* paralogs might have rendered one (or some) of the duplicated genes to have weaker purifying selection and experienced multiple mutations^44^. This could have caused non-silent changes in the outer membrane receptor and increased efficiency in interaction between bacterial and mammalian receptors. As a result, neofunctionalization of paralog could have happened and facilitated the emergence of *ail*.

Our study suggests that there is a possibility of lateral transfer of the *ail* gene from *Y. pseudotuberculosis* to *Y. enterocolitica*, supported by the higher percentage of protein sequence identity between *ail* from *Y. enterocolitica* and *ail* (and *ail* homologs) from *Y. pseudotuberculosis* compared to *ail* homolog from *Y. enterocolitica*. To the best of our knowledge, pYV (or pCD1) virulence plasmid is only present in human pathogenic *Yersinia*, but not apathogenic *Yersinia* species. As our data clearly showed that the virulence plasmids borne by the human pathogenic *Y. pseudotuberculosis* and *Y. enterocolitica* are generally highly similar, they might have the same origin. Since both *Y. pseudotuberculosis* and *Y. enterocolitica* are distantly related to each other and do not share the same direct ancestor, we propose that the virulence plasmids might have been transferred laterally, for example, from the *Y. pseudotuberculosis* to *Y. enterocolitica*. We believe that the transfer of the virulence plasmid from the *Y. enterocolitica* to *Y. pseudotuberculosis* is unlikely to happen. It is because the spacer in the CRISPR array of the *Y. pseudotuberculosis* are highly similar to the spacer-recognized region in the pYV plasmid of *Y. enterocolitica*, therefore *Y. pseudotuberculosis* could recognize and fragment the pYV plasmid from the *Y. enterocolitica*.

Our study suggests that the evolution of human pathogenic *Yersinia* species might not be completely in parallel or independent to each other^9^,^10^, but instead, there might be also some lateral gene transfer events. The evolution of pathogenic *Yersinia* might reach another milestone when *Y. pseudotuberculosis* evolved into *Y. pestis*, which is transmitted by flea^4^. This breakthrough was accompanied by the acquisition of pFra and pPst plasmids in the *Y. pestis*. The pFra and pPst plasmids are known to be important for transmission of flea-borne infection rather than food-borne^4^. The pFra plasmid encodes *ymt*, which enables *Y. pestis* to survive inside flea and ensure successful transmission to the infected hosts, while the pPst plasmid encodes for a Pla protein, which is an important virulence factor that causes systematic dissemination after *Y. pestis* is injected subcutaneously^4^.

Last but not least, our data showed that the metabolism genes and virulence genes known to be involved in pathogenicity, are also conserved in the apathogenic *Y. similis*^45^. These genes (*rip*ABC, *ter*ZABCD, *pil* locus, *ail* and *inv* homologs) are present in *Y. pseudotuberculosis* group and function to metabolize anti-microbial compounds (*rip*), persist in macrophages (*ter*) and contribute to pathogenicity^29^,^30^. The presence of these genes in the apathogenic *Y. similis* may make it prudent to monitor this species and its potential pathogenicity in different environmental situations in future.

**Figure 4:**
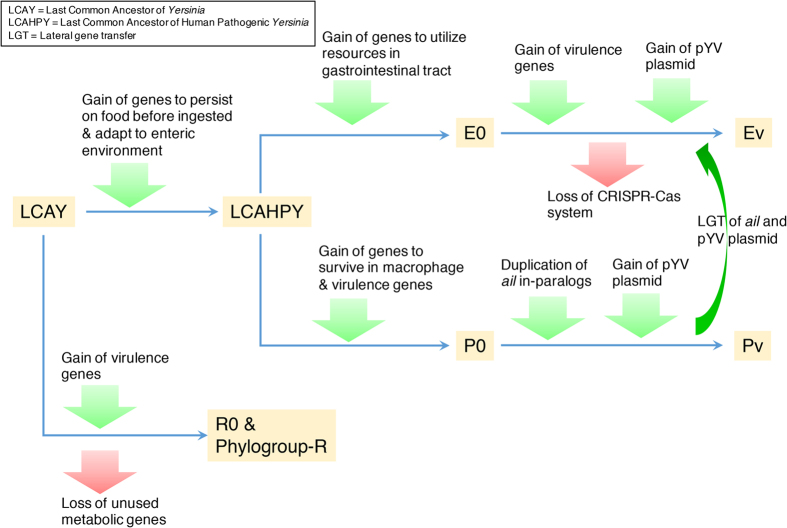
Proposed key evolutionary events that occurred in *Yersinia* and led to the emergence of pathogenic species. All hypothetical ancestors are highlighted in orange color and correspond to node in Fig. 2. Green and red colors indicate gene gain and gene loss respectively.

Based on our analyses, we hypothesize five possible main evolutionary events, in chorological order, to explain the emergence of human pathogenic *Yersinia* (Fig. 4).

1. LCAHPY might have, through gene gains, developed the ability to persist on food before being ingested, and adapted to gastrointestinal environment.
2. Diversification of *Yersinia* was likely to have occurred in new ecological niches by developing abilities to metabolize different nutrients available in different niches and body parts.
3. The gain of *ail* and *inv* genes might help the evolved species to adhere to and invade intestinal cell lining. *Y. pseudotuberculosis* might have gained *ail* through gene duplication and became donor of the gene to *Y. enterocolitica*.
4. Loss of CRISPR-Cas system and immunity against pYV virulence plasmid in some hypothetical ancestors.
5. Acquisition and maintenance of pYV virulence plasmid, followed by transformation into pathogenic species.

## Conclusion

Here we present an evolutionary study of human pathogenic *Yersinia* species. In contrast to previous studies^9^,^10^, we found that the evolution of the *Y. enterocolitica* and *Y. pseudotuberculosis*/*Y. pestis* might not be totally parallel. Instead, some of the virulence loci might have been transferred laterally from *Y. pseudotuberculosis*/*Y. pestis* to *Y. enterocolitica*. In summary, our study provides better insights into the evolution of human pathogenic *Yersinia*.

## Method

### Genome sequences and annotation

A total of 124 complete genome sequences of *Enterobacteriaceae* (including 15 *Yersinia* species) and 2 *Haemophilus influenza* were downloaded from National Center for Biotechnology Information (NCBI) database^1^. Details of *Yersinia* genomes are tabulated in Supplementary Table 1. For consistency, all genomes were annotated using Rapid Annotation using Subsystem Technology (RAST) online server to generate a list of open reading frames (ORFs) and protein sequences (see Supplementary Table 2 for summary)^46^. Then, the function of each protein sequence was predicted by using BLASTP to search for homolog in COG (E-value cutoff: 1E-5), KOBAS (default cutoff) and Virulence Factors Database (E-value cutoff: 1E-5) while HMMER was used to search against TIGRFAM^47^,^48^,^49^,^50^,^51^,^52^.

JSpecies was used to calculate average nucleotide identity (ANI) value between *Yersinia* pYV plasmids^53^.

### Protein sequences clustering

All protein sequences were clustered thrice by using ProteinOrtho with default parameters^54^ (E-value cutoff: 1E-5; minimum percentage of identity: 25%; and minimum percentage of coverage: 50%). The first dataset consisted of all Enterobacteriaceae and *Haemophilus influenza* (hereinafter named the Enterobacteriaceae dataset), the second dataset consisted of *Serratia liquefaciens* and all *Yersinia* (hereinafter named the *Yersinia*-*Serratia* dataset) and the third dataset consisted of all *Yersinia* (hereinafter named the *Yersinia* dataset).

### Multiple sequence alignment

Protein sequences of single copy core genes from all datasets were aligned using L-INS-i algorithm implemented in Multiple Alignment using Fast Fourier Transform (MAFFT) program^55^. Then, aligned protein sequences of each gene family were translated back to codon alignment using PAL2NAL^56^, and poorly aligned region was removed using GBlocks^57^.

### Recombination testing

Codon alignments of the *Yersinia*-*Serratia* and Enterobacteriaceae datasets were used as input to PHI to test for recombination with 10,000 iterations and 0.05 as p-value cutoff 58. Next, alignments without recombination were concatenated together to form a “super-sequence” in the two dataset independently. ClonalFrameML was used to estimate rate of recombination to mutation in *Yersinia* dataset^59^.

### Phylogenetic tree construction

Super-sequence of the *Yersinia*-*Serratia* dataset was used to infer phylogenetic trees using RAxML^60^, with maximum likelihood method, GTR + GAMMA model and 1,000 bootstrap iterations. Due to the large Enterobacteriaceae dataset, the Enterobacteriaceae phylogenetic tree was constructed by maximum likelihood in FastTree2 with 1,000 bootstrap iterations^61^. A matrix consisting of presence and absence of gene family in *Yersinia*-*Serratia* dataset was used to construct the gene content phylogenetic tree using neighbor-joining implemented in MEGA6^11^,^62^.

### Gene gain-and-loss analysis

Reconstruction of gene gain-and-loss in *Yersinia* genus was performed using Enterobacteriaceae dataset and COUNT with 1.5 as gain penalty^63^. Then, acquired and lost pathways and genes in ancestors of interest were inspected manually.

### CRISPR analysis

CRISPR was predicted using CRT^64^. The spacer within CRISPR array was then searched against NCBI database using BLASTN to look for closely related plasmid sequence.

### Virulence *ail* and *inv* genes analysis

Protein sequences of functional *ail* from *Y. pestis* CO92 and *inv* from *Y. enterocolitica* 8081 were used to search for their respective homologs in *Yersinia* using BLASTP^47^. The BLASTP outputs were further filtered by 1E-7 for E-value, 50% sequence completeness for subject and query sequences. All putative *ail* homologs were searched against NCBI NR database, and functional *ail* of *Y. enterocolitica* was searched against *ail* homologs of *Yersinia* using BLASTP^47^.

## Additional Information

**How to cite this article**: Tan, S. Y. *et al*. Evolutionary study of *Yersinia* genomes deciphers emergence of human pathogenic species. *Sci. Rep.* **6**, 36116; doi: 10.1038/srep36116 (2016).

**Publisher’s note:** Springer Nature remains neutral with regard to jurisdictional claims in published maps and institutional affiliations.

## Supplementary Material

Supplementary Information

## References

[b1] Benson D. A. . GenBank. Nucleic Acids Res 43, D30–D35, 10.1093/nar/gku1216 (2015). 25414350 PMC4383990

[b2] Wren B. W. The yersiniae—a model genus to study the rapid evolution of bacterial pathogens. Nat Rev Microbiol 1, 55–64, 10.1038/nrmicro730 (2003). 15040180

[b3] Sulakvelidze A. Yersiniae other than Y. enterocolitica, Y. pseudotuberculosis, and Y. pestis: the ignored species. Microbes Infect 2, 497–513 (2000). 10865195 10.1016/s1286-4579(00)00311-7

[b4] Achtman M. . Yersinia pestis, the cause of plague, is a recently emerged clone of Yersinia pseudotuberculosis. Proc Natl Acad Sci USA 96, 14043–14048 (1999). 10570195 10.1073/pnas.96.24.14043 PMC24187

[b5] Cornelis G. R. The Yersinia Ysc-Yop ‘type III’ weaponry. Nat Rev Mol Cell Biol 3, 742–752, 10.1038/nrm932 (2002). 12360191

[b6] Mikula K. M., Kolodziejczyk R. & Goldman A. Yersinia infection tools-characterization of structure and function of adhesins. Front Cell Infect Microbiol 2, 169, 10.3389/fcimb.2012.00169 (2012). 23316485 PMC3539135

[b7] Perry R. D. & Fetherston J. D. Yersinia pestis--etiologic agent of plague. Clin Microbiol Rev 10, 35–66 (1997). 8993858 10.1128/cmr.10.1.35 PMC172914

[b8] Bottone E. J. Yersinia enterocolitica: the charisma continues. Clin Microbiol Rev 10, 257–276 (1997). 9105754 10.1128/cmr.10.2.257 PMC172919

[b9] Reuter S. . Parallel independent evolution of pathogenicity within the genus Yersinia. Proc Natl Acad Sci USA 111, 6768–6773, 10.1073/pnas.1317161111 (2014). 24753568 PMC4020045

[b10] McNally A., Thomson N. R., Reuter S. & Wren B. W. ‘Add, stir and reduce’: Yersinia spp. as model bacteria for pathogen evolution. Nat Rev Microbiol 14, 177–190, 10.1038/nrmicro.2015.29 (2016). 26876035

[b11] Snel B., Bork P. & Huynen M. A. Genome phylogeny based on gene content. Nat Genet 21, 108–110, 10.1038/5052 (1999). 9916801

[b12] Majewski J., Zawadzki P., Pickerill P., Cohan F. M. & Dowson C. G. Barriers to genetic exchange between bacterial species: Streptococcus pneumoniae transformation. J Bacteriol 182, 1016–1023 (2000). 10648528 10.1128/jb.182.4.1016-1023.2000 PMC94378

[b13] Brocker M., Schaffer S., Mack C. & Bott M. Citrate utilization by Corynebacterium glutamicum is controlled by the CitAB two-component system through positive regulation of the citrate transport genes citH and tctCBA. J Bacteriol 191, 3869–3880, 10.1128/JB.00113-09 (2009). 19376865 PMC2698389

[b14] Letoffe S., Nato F., Goldberg M. E. & Wandersman C. Interactions of HasA, a bacterial haemophore, with haemoglobin and with its outer membrane receptor HasR. Mol Microbiol 33, 546–555 (1999). 10417645 10.1046/j.1365-2958.1999.01499.x

[b15] Connolly J. P. . A Highly Conserved Bacterial D-Serine Uptake System Links Host Metabolism and Virulence. PLoS Pathog 12, e1005359, 10.1371/journal.ppat.1005359 (2016). 26727373 PMC4699771

[b16] Haller J. C., Carlson S., Pederson K. J. & Pierson D. E. A chromosomally encoded type III secretion pathway in Yersinia enterocolitica is important in virulence. Mol Microbiol 36, 1436–1446 (2000). 10931293 10.1046/j.1365-2958.2000.01964.x

[b17] Iwobi A. . Novel virulence-associated type II secretion system unique to high-pathogenicity Yersinia enterocolitica. Infect Immun 71, 1872–1879 (2003). 12654803 10.1128/IAI.71.4.1872-1879.2003 PMC152056

[b18] Fernandez L., Marquez I. & Guijarro J. A. Identification of specific *in vivo*-induced (ivi) genes in Yersinia ruckeri and analysis of ruckerbactin, a catecholate siderophore iron acquisition system. Appl Environ Microbiol 70, 5199–5207, 10.1128/AEM.70.9.5199-5207.2004 (2004). 15345400 PMC520893

[b19] Lee E. J., Cho Y. H., Kim H. S., Ahn B. E. & Roe J. H. Regulation of sigmaB by an anti- and an anti-anti-sigma factor in Streptomyces coelicolor in response to osmotic stress. J Bacteriol 186, 8490–8498, 10.1128/JB.186.24.8490-8498.2004 (2004). 15576799 PMC532406

[b20] Cao J., Woodhall M. R., Alvarez J., Cartron M. L. & Andrews S. C. EfeUOB (YcdNOB) is a tripartite, acid-induced and CpxAR-regulated, low-pH Fe2+ transporter that is cryptic in Escherichia coli K-12 but functional in *Escherichia coli* O157:H7. Mol Microbiol 65, 857–875, 10.1111/j.1365-2958.2007.05802.x (2007). 17627767

[b21] Yaron S. & Romling U. Biofilm formation by enteric pathogens and its role in plant colonization and persistence. Microb Biotechnol 7, 496–516, 10.1111/1751-7915.12186 (2014). 25351039 PMC4265070

[b22] Roy C. . Modes of action of five different endopectate lyases from Erwinia chrysanthemi 3937. J Bacteriol 181, 3705–3709 (1999). 10368144 10.1128/jb.181.12.3705-3709.1999 PMC93847

[b23] Hugouvieux-Cotte-Pattat N. & Reverchon S. Two transporters, TogT and TogMNAB, are responsible for oligogalacturonide uptake in Erwinia chrysanthemi 3937. Mol Microbiol 41, 1125–1132 (2001). 11555292 10.1046/j.1365-2958.2001.02565.x

[b24] Yamazaki A. . Commensal effect of pectate lyases secreted from Dickeya dadantii on proliferation of Escherichia coli O157:H7 EDL933 on lettuce leaves. Appl Environ Microbiol 77, 156–162, 10.1128/AEM.01079-10 (2011). 21075884 PMC3019694

[b25] Mobley H. L. The role of Helicobacter pylori urease in the pathogenesis of gastritis and peptic ulceration. Aliment Pharmacol Ther 10 Suppl 1, 57–64 (1996). 8730260 10.1046/j.1365-2036.1996.22164006.x

[b26] Xavier K. B. . Phosphorylation and processing of the quorum-sensing molecule autoinducer-2 in enteric bacteria. ACS Chem Biol 2, 128–136, 10.1021/cb600444h (2007). 17274596

[b27] Rohmer L., Hocquet D. & Miller S. I. Are pathogenic bacteria just looking for food? Metabolism and microbial pathogenesis. Trends Microbiol 19, 341–348, 10.1016/j.tim.2011.04.003 (2011). 21600774 PMC3130110

[b28] Ponnusamy D. & Clinkenbeard K. D. Role of Tellurite Resistance Operon in Filamentous Growth of Yersinia pestis in Macrophages. PLoS One 10, e0141984, 10.1371/journal.pone.0141984 (2015). 26536670 PMC4633105

[b29] Ponnusamy D., Hartson S. D. & Clinkenbeard K. D. Intracellular Yersinia pestis expresses general stress response and tellurite resistance proteins in mouse macrophages. Vet Microbiol 150, 146–151, 10.1016/j.vetmic.2010.12.025 (2011). 21295415

[b30] Sasikaran J., Ziemski M., Zadora P. K., Fleig A. & Berg I. A. Bacterial itaconate degradation promotes pathogenicity. Nat Chem Biol 10, 371–377, 10.1038/nchembio.1482 (2014). 24657929

[b31] Collyn F. . Yersinia pseudotuberculosis harbors a type IV pilus gene cluster that contributes to pathogenicity. Infect Immun 70, 6196–6205 (2002). 12379698 10.1128/IAI.70.11.6196-6205.2002 PMC130390

[b32] Yang Y., Merriam J. J., Mueller J. P. & Isberg R. R. The psa locus is responsible for thermoinducible binding of Yersinia pseudotuberculosis to cultured cells. Infect Immun 64, 2483–2489 (1996). 8698470 10.1128/iai.64.7.2483-2489.1996 PMC174101

[b33] Pontes M. H., Lee E. J., Choi J. & Groisman E. A. Salmonella promotes virulence by repressing cellulose production. Proc Natl Acad Sci USA 112, 5183–5188, 10.1073/pnas.1500989112 (2015). 25848006 PMC4413311

[b34] Brown B. L. . Three dimensional structure of the MqsR:MqsA complex: a novel TA pair comprised of a toxin homologous to RelE and an antitoxin with unique properties. PLoS Pathog 5, e1000706, 10.1371/journal.ppat.1000706 (2009). 20041169 PMC2791442

[b35] Schubert S., Rakin A. & Heesemann J. The Yersinia high-pathogenicity island (HPI): evolutionary and functional aspects. Int J Med Microbiol 294, 83–94, 10.1016/j.ijmm.2004.06.026 (2004). 15493818

[b36] Ravenhall M., Skunca N., Lassalle F. & Dessimoz C. Inferring horizontal gene transfer. PLoS Comput Biol 11, e1004095, 10.1371/journal.pcbi.1004095 (2015). 26020646 PMC4462595

[b37] Leong J. M., Fournier R. S. & Isberg R. R. Identification of the integrin binding domain of the Yersinia pseudotuberculosis invasin protein. EMBO J 9, 1979–1989 (1990). 1693333 10.1002/j.1460-2075.1990.tb08326.x PMC551907

[b38] Haft D. H., Selengut J., Mongodin E. F. & Nelson K. E. A guild of 45 CRISPR-associated (Cas) protein families and multiple CRISPR/Cas subtypes exist in prokaryotic genomes. PLoS Comput Biol 1, e60, 10.1371/journal.pcbi.0010060 (2005). 16292354 PMC1282333

[b39] Hammerl J. A., Klein I., Lanka E., Appel B. & Hertwig S. Genetic and functional properties of the self-transmissible Yersinia enterocolitica plasmid pYE854, which mobilizes the virulence plasmid pYV. J Bacteriol 190, 991–1010, 10.1128/JB.01467-07 (2008). 18055592 PMC2223581

[b40] Lightfield J., Fram N. R. & Ely B. Across bacterial phyla, distantly-related genomes with similar genomic GC content have similar patterns of amino acid usage. PLoS One 6, e17677, 10.1371/journal.pone.0017677 (2011). 21423704 PMC3053387

[b41] Neyt C., Iriarte M., Thi V. H. & Cornelis G. R. Virulence and arsenic resistance in Yersiniae. J Bacteriol 179, 612–619 (1997). 9006011 10.1128/jb.179.3.612-619.1997 PMC178738

[b42] Lassalle F., Muller D. & Nesme X. Ecological speciation in bacteria: reverse ecology approaches reveal the adaptive part of bacterial cladogenesis. Res Microbiol 166, 729–741, 10.1016/j.resmic.2015.06.008 (2015). 26192210

[b43] San Millan A. . Positive selection and compensatory adaptation interact to stabilize non-transmissible plasmids. Nat Commun 5, 5208, 10.1038/ncomms6208 (2014). 25302567 PMC4208098

[b44] Kondrashov F. A., Rogozin I. B., Wolf Y. I. & Koonin E. V. Selection in the evolution of gene duplications. Genome Biol **3**, RESEARCH0008 (2002). 10.1186/gb-2002-3-2-research0008 PMC65685 11864370

[b45] Sprague L. D. & Neubauer H. Genome Sequence of Yersinia similis Y228T, a Member of the Yersinia pseudotuberculosis Complex. Genome Announc 2, 10.1128/genomeA.00216-14 (2014). PMC3968338 24675860

[b46] Aziz R. K. . The RAST Server: rapid annotations using subsystems technology. BMC Genomics 9, 75, 10.1186/1471-2164-9-75 (2008). 18261238 PMC2265698

[b47] Altschul S. F., Gish W., Miller W., Myers E. W. & Lipman D. J. Basic local alignment search tool. J Mol Biol 215, 403–410, 10.1016/S0022-2836(05)80360-2 (1990). 2231712

[b48] Galperin M. Y., Makarova K. S., Wolf Y. I. & Koonin E. V. Expanded microbial genome coverage and improved protein family annotation in the COG database. Nucleic Acids Res 43, D261–D269, 10.1093/nar/gku1223 (2015). 25428365 PMC4383993

[b49] Xie C. . KOBAS 2.0: a web server for annotation and identification of enriched pathways and diseases. Nucleic Acids Res 39, W316–W322, 10.1093/nar/gkr483 (2011). 21715386 PMC3125809

[b50] Chen L., Xiong Z., Sun L., Yang J. & Jin Q. VFDB 2012 update: toward the genetic diversity and molecular evolution of bacterial virulence factors. Nucleic Acids Res 40, D641–D645, 10.1093/nar/gkr989 (2012). 22067448 PMC3245122

[b51] Eddy S. R. A new generation of homology search tools based on probabilistic inference. Genome Inform 23, 205–211 (2009). 20180275

[b52] Haft D. H., Selengut J. D. & White O. The TIGRFAMs database of protein families. Nucleic Acids Res 31, 371–373 (2003). 12520025 10.1093/nar/gkg128 PMC165575

[b53] Richter M. & Rossello-Mora R. Shifting the genomic gold standard for the prokaryotic species definition. Proc Natl Acad Sci USA 106, 19126–19131, 10.1073/pnas.0906412106 (2009). 19855009 PMC2776425

[b54] Lechner M. . Proteinortho: detection of (co-)orthologs in large-scale analysis. BMC Bioinformatics 12, 124, 10.1186/1471-2105-12-124 (2011). 21526987 PMC3114741

[b55] Katoh K. & Standley D. M. MAFFT multiple sequence alignment software version 7: improvements in performance and usability. Mol Biol Evol 30, 772–780, 10.1093/molbev/mst010 (2013). 23329690 PMC3603318

[b56] Suyama M., Torrents D. & Bork P. PAL2NAL: robust conversion of protein sequence alignments into the corresponding codon alignments. Nucleic Acids Res 34, W609–W612, 10.1093/nar/gkl315 (2006). 16845082 PMC1538804

[b57] Talavera G. & Castresana J. Improvement of phylogenies after removing divergent and ambiguously aligned blocks from protein sequence alignments. Syst Biol 56, 564–577, 10.1080/10635150701472164 (2007). 17654362

[b58] Bruen T. C., Philippe H. & Bryant D. A simple and robust statistical test for detecting the presence of recombination. Genetics 172, 2665–2681, 10.1534/genetics.105.048975 (2006). 16489234 PMC1456386

[b59] Didelot X. & Wilson D. J. ClonalFrameML: efficient inference of recombination in whole bacterial genomes. PLoS Comput Biol 11, e1004041, 10.1371/journal.pcbi.1004041 (2015). 25675341 PMC4326465

[b60] Stamatakis A. RAxML version 8: a tool for phylogenetic analysis and post-analysis of large phylogenies. Bioinformatics 30, 1312–1313, 10.1093/bioinformatics/btu033 (2014). 24451623 PMC3998144

[b61] Price M. N., Dehal P. S. & Arkin A. P. FastTree 2–approximately maximum-likelihood trees for large alignments. PLoS One 5, e9490, 10.1371/journal.pone.0009490 (2010). 20224823 PMC2835736

[b62] Tamura K., Stecher G., Peterson D., Filipski A. & Kumar S. MEGA6: Molecular Evolutionary Genetics Analysis version 6.0. Mol Biol Evol 30, 2725–2729, 10.1093/molbev/mst197 (2013). 24132122 PMC3840312

[b63] Csuros M. Count: evolutionary analysis of phylogenetic profiles with parsimony and likelihood. Bioinformatics 26, 1910–1912, 10.1093/bioinformatics/btq315 (2010). 20551134

[b64] Bland C. . CRISPR recognition tool (CRT): a tool for automatic detection of clustered regularly interspaced palindromic repeats. BMC Bioinformatics 8, 209, 10.1186/1471-2105-8-209 (2007). 17577412 PMC1924867

