# Supplementary material for: Evolutionary study of Yersinia genomes deciphers emergence of human pathogenic species
Source: Sci Rep. 2016 Oct 31;6:36116. doi: 10.1038/srep36116 (PMC5086877; doi:10.1038/srep36116)
Supplement: Supplementary Information [file srep36116-s1.doc]

**Evolutionary study of *Yersinia* genomes deciphers emergence of human pathogenic species**

Shi Yang Tan^1,2^, Irene Kit Ping Tan^3^, Mui Fern Tan^2^, Avirup Dutta^2^, Siew Woh Choo^1,2^

^1^Department of Oral and Craniofacial Sciences, Faculty of Dentistry, University of Malaya, 50603 Kuala Lumpur, Malaysia.

^2^Genome Informatics Research Laboratory, High Impact Research Building, University of Malaya, 50603 Kuala Lumpur, Malaysia.

^3^Institute of Biological Sciences, Faculty of Science, University of Malaya, 50603 Kuala Lumpur, Malaysia.

Corresponding author=SWC, IKPT

SYT shiyangtan@gmail.com

IKPT itan@um.edu.my

MFT muifern007@gmail.com

AD avirupdutta@gmail.com

SWC l.choo@genomesolutions.com.my

# List of supplementary figures

Supplementary Figure 1: *Yersinia* gene content phylogenetic tree constructed using presence and absence of gene in each family, and rooted using *S. liquefaciens*. Phylogroup-P, phylogroup-E, and phylogroup-R are highlighted in magenta, cyan, and yellow respectively. Last Common Ancestor of all *Yersinia* (LCAY) is hypothesized as the most recent hypothetical ancestor shared by all *Yersinia* species while Last Common Ancestor of Human Pathogenic *Yersinia* (LCAHPY) is hypothesized as the most recent hypothetical ancestor shared by human virulent *Y. enterocolitica*, *Y. pseudotuberculosis*, and *Y. pestis*.


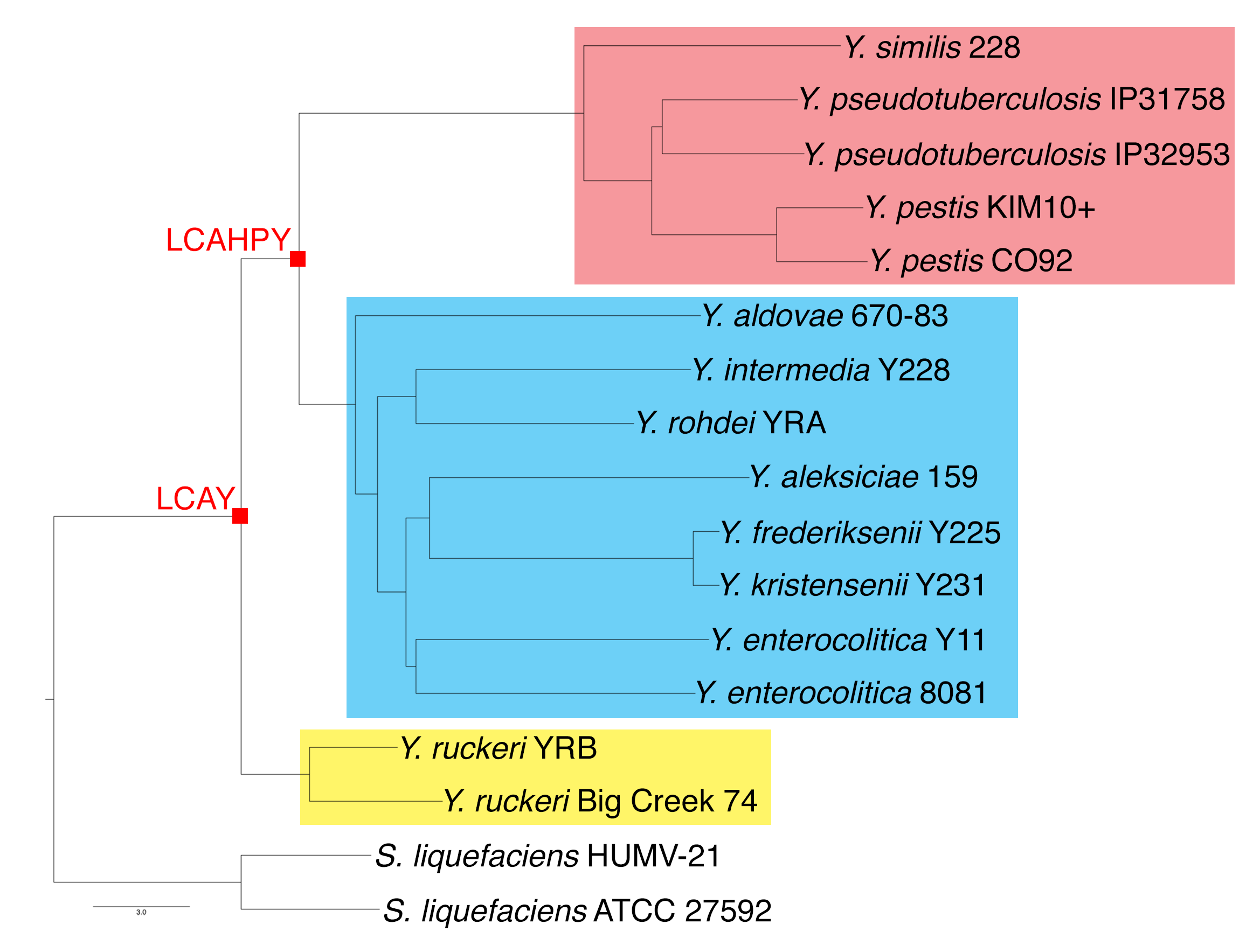


# List of supplementary tables

Supplementary Table 1: List of *Yersinia* genome sequences used in this study with their corresponding isolation sources and geographical area. Human virulent strain is colored in red.

| **Species** | **Strain** | **NCBI Accession** | **Isolation source** | **Geographic location** |
| --- | --- | --- | --- | --- |
| *Yersinia aldovae* | 670-83 | CP009781.1 | Fish | Norway |
| *Yersinia aleksiciae* | 159 | CP011975.1 | Human faeces | Finland |
| *Yersinia enterocolitica* | Y11 | FR729477.2 | Stool of human patient | Germany |
| *Yersinia enterocolitica* | 8081 | AM286415.1 | Human (with fatal septicaemia) | Ohio, United States |
| *Yersinia frederiksenii* | Y225 | CP009364.1 | N/A | N/A |
| *Yersinia intermedia* | Y228 | CP009801.1 | N/A | N/A |
| *Yersinia kristensenii* | Y231 | CP009997.1 | N/A | N/A |
| *Yersinia pestis* | KIM10 | AE009952.1 | Human (pneumonic plague) | Kurdistan, Iran |
| *Yersinia pestis* | CO92 | AL590842.1 | Human (fatal pneumonic plague) | United States |
| *Yersinia pseudotuberculosis* | IP31758 | CP000720.1 | Human patient | Primorski, Soviet Union |
| *Yersinia pseudotuberculosis* | IP32953 | BX936398.1 | Human patient | France |
| *Yersinia rohdei* | YRA | CP009787.1 | Animal faeces | Germany |
| *Yersinia ruckeri* | YRB | CP009539.1 | Fish (liver tissue) | N/A |
| *Yersinia ruckeri* | Big Creek 74 | CP011078.1 | *Oncorhynchus tshawytscha* | Oregon, United States |
| *Yersinia similis* | 228 | CP007230.1 | Rabbit | Germany |

Supplementary Table 2: Summary of genome annotation of *Yersinia* genomes used in this study. Known human virulent strain is colored in red.

| **Species** | **Strain** | **Genome size (bp)** | **GC content (%)** | **Total CDS** | **Total rRNA operon** | **Total tRNA** |
| --- | --- | --- | --- | --- | --- | --- |
| *Yersinia aldovae* | 670-83 | 4,471,090 | 47.69 | 3,985 | 7 | 82 |
| *Yersinia aleksiciae* | 159 | 4,000,307 | 49.04 | 3,569 | 7 | 75 |
| *Yersinia enterocolitica* | Y11 | 4,553,420 | 47.01 | 4,155 | 7 | 70 |
| *Yersinia enterocolitica* | 8081 | 4,615,899 | 47.27 | 4,167 | 7 | 81 |
| *Yersinia frederiksenii* | Y225 | 4,495,532 | 47.40 | 4,016 | 7 | 82 |
| *Yersinia intermedia* | Y228 | 4,859,749 | 47.47 | 4,320 | 7 | 81 |
| *Yersinia kristensenii* | Y231 | 4,496,569 | 47.40 | 4,012 | 7 | 81 |
| *Yersinia pestis* | KIM10 | 4,600,755 | 47.64 | 4,033 | 7 | 73 |
| *Yersinia pestis* | CO92 | 4,653,728 | 47.64 | 4,090 | 6 | 70 |
| *Yersinia pseudotuberculosis* | IP31758 | 4,723,306 | 47.54 | 4,013 | 7 | 86 |
| *Yersinia pseudotuberculosis* | IP32953 | 4,743,972 | 47.61 | 4,072 | 7 | 85 |
| *Yersinia rohdei* | YRA | 4,372,253 | 47.03 | 3,791 | 7 | 81 |
| *Yersinia ruckeri* | YRB | 3,605,216 | 47.50 | 3,162 | 7 | 79 |
| *Yersinia ruckeri* | Big Creek 74 | 3,699,725 | 47.64 | 3,268 | 7 | 81 |
| *Yersinia similis* | 228 | 4,903,722 | 46.97 | 4,327 | 7 | 87 |

Supplementary Table 3: Cluster of gene homologous to reference *ail* (VFDB identifier: VFG0354, form *Y. pestis* CO92) in *Yersinia* and the respective BLASTP output. Known *ail* of human virulent species is highlighted in grey.

| **Cluster** | **Genome** | **Locus tag** | **Query length** | **Query start** | **Query end** | **Subject start** | **Subject end** | **Query coverage (%)** | **Identity (%)** | **Alignment Length** |
| --- | --- | --- | --- | --- | --- | --- | --- | --- | --- | --- |
| 1 | *Y. similis* 228 | CP007230.1_CDS_3282 | 184 | 1 | 184 | 4 | 182 | 100.00 | 42.70 | 185 |
|  | *Y. pseudotuberculosis* IP31758 | CP000720.1_CDS_2171 | 183 | 3 | 183 | 5 | 182 | 98.91 | 45.36 | 183 |
|  |  | CP000720.1_CDS_1869 | 179 | 6 | 179 | 7 | 182 | 97.21 | 44.94 | 178 |
|  |  | CP000720.1_CDS_1114 | 179 | 1 | 179 | 4 | 182 | 100.00 | 99.44 | 179 |
|  | *Y. pseudotuberculosis* IP32953 | BX936398.1_CDS_2930 | 179 | 1 | 179 | 4 | 182 | 100.00 | 99.44 | 179 |
|  |  | BX936398.1_CDS_1757 | 183 | 3 | 183 | 5 | 182 | 98.91 | 45.36 | 183 |
|  |  | BX936398.1_CDS_2167 | 179 | 6 | 179 | 7 | 182 | 97.21 | 44.94 | 178 |
|  | *Y. pestis* CO92 | AL590842.1_CDS_1847 | 183 | 3 | 183 | 5 | 182 | 98.91 | 45.36 | 183 |
|  |  | AL590842.1_CDS_2879 | 179 | 1 | 179 | 4 | 182 | 100.00 | 100.00 | 179 |
|  |  | AL590842.1_CDS_2180 | 179 | 6 | 179 | 7 | 182 | 97.21 | 44.94 | 178 |
|  | *Y. pestis* KIM10+ | AE009952.1_CDS_1968 | 179 | 6 | 179 | 7 | 182 | 97.21 | 44.94 | 178 |
|  |  | AE009952.1_CDS_2392 | 183 | 3 | 183 | 5 | 182 | 98.91 | 45.36 | 183 |
|  |  | AE009952.1_CDS_1299 | 179 | 1 | 179 | 4 | 182 | 100.00 | 100.00 | 179 |
| 2 | *Y. enterocolitica* Y11 | FR729477.2_CDS_21 | 178 | 1 | 178 | 4 | 182 | 100.00 | 74.30 | 179 |
|  | *Y. enterocolitica* 8081 | AM286415.1_CDS_1784 | 178 | 1 | 178 | 4 | 182 | 100.00 | 73.74 | 179 |
|  | *Y. similis* 228 | CP007230.1_CDS_63 | 179 | 1 | 179 | 4 | 182 | 100.00 | 91.62 | 179 |
|  |  | CP007230.1_CDS_1815 | 178 | 1 | 178 | 4 | 182 | 100.00 | 60.89 | 179 |
| 3 | *Y. aldovae* 670-83 | CP009781.1_CDS_3803 | 175 | 1 | 175 | 4 | 182 | 100.00 | 39.56 | 182 |
|  | *Y. intermedia* Y228 | CP009801.1_CDS_3158 | 175 | 1 | 175 | 4 | 182 | 100.00 | 38.25 | 183 |
|  | *Y. aleksiciae* 159 | CP011975.1_CDS_443 | 175 | 1 | 175 | 4 | 182 | 100.00 | 38.92 | 185 |
|  | *Y. rohdei* YRA | CP009787.1_CDS_3781 | 175 | 1 | 175 | 4 | 182 | 100.00 | 38.80 | 183 |
|  | *Y. frederiksenii* Y225 | CP009364.1_CDS_591 | 175 | 1 | 175 | 4 | 182 | 100.00 | 38.92 | 185 |
|  | *Y. kristensenii* Y231 | CP009997.1_CDS_1027 | 175 | 1 | 175 | 4 | 182 | 100.00 | 38.92 | 185 |
|  | *Y. enterocolitica* Y11 | FR729477.2_CDS_1664 | 175 | 1 | 175 | 4 | 182 | 100.00 | 39.46 | 185 |
|  | *Y. enterocolitica* 8081 | AM286415.1_CDS_2785 | 175 | 1 | 175 | 4 | 182 | 100.00 | 39.46 | 185 |
|  | *Y. similis* 228 | CP007230.1_CDS_4080 | 174 | 1 | 174 | 4 | 182 | 100.00 | 40.66 | 182 |
|  | *Y. pseudotuberculosis* IP31758 | CP000720.1_CDS_1436 | 174 | 1 | 174 | 4 | 182 | 100.00 | 40.66 | 182 |
|  | *Y. pseudotuberculosis* IP32953 | BX936398.1_CDS_2607 | 174 | 1 | 174 | 4 | 182 | 100.00 | 40.66 | 182 |
|  | *Y. pestis* CO92 | AL590842.1_CDS_2487 | 174 | 1 | 174 | 4 | 182 | 100.00 | 40.66 | 182 |
|  | *Y. pestis* KIM10+ | AE009952.1_CDS_1634 | 174 | 1 | 174 | 4 | 182 | 100.00 | 40.66 | 182 |
|  | *Y. ruckeri* YRB | CP009539.1_CDS_3022 | 174 | 1 | 174 | 4 | 182 | 100.00 | 39.13 | 184 |
|  | *Y. ruckeri* Big Creek 74 | CP011078.1_CDS_3141 | 174 | 1 | 174 | 4 | 182 | 100.00 | 38.46 | 182 |

Supplementary Table 4: BLASTP output of functional *ail* in *Y. enterocolitica* 8081 which was used as query to search against *ail* homologs in *Yersinia*. Phylogroup-P species, which are highlighted in grey, were in the top significant hits.

| Genome name | Subject ID | Identity (%) | Alignment length | E-value | Bit score |
| --- | --- | --- | --- | --- | --- |
| *Y. similis* 228 | CP007230.1_CDS_63 | 74.86 | 179 | 3.72E-97 | 269 |
| *Y. pseudotuberculosis* IP32953 | BX936398.1_CDS_2930 | 73.74 | 179 | 1.10E-93 | 261 |
| *Y. pseudotuberculosis* IP31758 | CP000720.1_CDS_1114 | 73.74 | 179 | 1.10E-93 | 261 |
| *Y. similis* 228 | CP007230.1_CDS_1815 | 66.85 | 178 | 1.09E-90 | 253 |
| *Y. pseudotuberculosis* IP31758 | CP000720.1_CDS_2171 | 48.90 | 182 | 9.26E-56 | 164 |
| *Y. pseudotuberculosis* IP32953 | BX936398.1_CDS_1757 | 46.74 | 184 | 9.67E-56 | 164 |
| *Y. similis* 228 | CP007230.1_CDS_3282 | 46.49 | 185 | 2.12E-55 | 164 |
| *Y. pseudotuberculosis* IP32953 | BX936398.1_CDS_2167 | 46.39 | 166 | 2.95E-51 | 153 |
| *Y. pseudotuberculosis* IP31758 | CP000720.1_CDS_1869 | 46.39 | 166 | 2.95E-51 | 153 |
| *Y. ruckeri* Big Creek 74 | CP011078.1_CDS_3141 | 40.33 | 181 | 2.23E-42 | 130 |
| *Y. ruckeri* YRB | CP009539.1_CDS_3022 | 40.33 | 181 | 7.85E-42 | 129 |
| *Y. aldovae* 670-83 | CP009781.1_CDS_3803 | 38.46 | 182 | 8.50E-39 | 121 |
| *Y. similis* 228 | CP007230.1_CDS_4080 | 38.30 | 188 | 9.44E-38 | 118 |
| *Y. pseudotuberculosis* IP32953 | BX936398.1_CDS_2607 | 38.30 | 188 | 1.57E-37 | 118 |
| *Y. pseudotuberculosis* IP31758 | CP000720.1_CDS_1436 | 38.30 | 188 | 1.57E-37 | 118 |
| *Y. intermedia* Y228 | CP009801.1_CDS_3158 | 37.36 | 182 | 1.80E-36 | 115 |
| *Y. kristensenii* Y231 | CP009997.1_CDS_1027 | 37.91 | 182 | 2.33E-36 | 115 |
| *Y. frederiksenii* Y225 | CP009364.1_CDS_591 | 37.91 | 182 | 2.33E-36 | 115 |
| *Y. enterocolitica* Y11 | FR729477.2_CDS_1664 | 36.81 | 182 | 2.41E-36 | 115 |
| *Y. enterocolitica* 8081 | AM286415.1_CDS_2785 | 36.81 | 182 | 2.41E-36 | 115 |
| *Y. rohdei* YRA | CP009787.1_CDS_3781 | 37.16 | 183 | 7.10E-36 | 114 |
| *Y. aleksiciae* 159 | CP011975.1_CDS_443 | 36.81 | 182 | 5.47E-35 | 111 |

Supplementary Table 5: BLASTP output where *inv* of *Y. enterocolitica* 8081 (with 835 amino acids) was used as reference query to search for homologs in *Yersinia*. Functional *inv* of human pathogenic species is highlighted in grey.

| Subject name | Subject ID | Subject start | Subject end | Subject length | Query start | Query end | E-value | Identity (%) |
| --- | --- | --- | --- | --- | --- | --- | --- | --- |
| *Y. aldovae* 670-83 | CP009781.1_CDS_159 | 58 | 811 | 850 | 46 | 808 | 3.00E-169 | 40.67 |
| *Y. aldovae* 670-83 | CP009781.1_CDS_407 | 159 | 619 | 828 | 69 | 540 | 4.00E-134 | 46.33 |
| *Y. enterocolitica* 8081 | AM286415.1_CDS_2507 | 1 | 835 | 835 | 1 | 835 | 0 | 100 |
| *Y. enterocolitica* Y11 | FR729477.2_CDS_1378 | 1 | 835 | 835 | 1 | 835 | 0 | 99.04 |
| *Y. frederiksenii* Y225 | CP009364.1_CDS_1102 | 131 | 653 | 1038 | 69 | 623 | 1.00E-89 | 36.56 |
| *Y. kristensenii* Y231 | CP009997.1_CDS_515 | 131 | 653 | 1038 | 69 | 623 | 1.00E-89 | 36.56 |
| *Y. pestis* CO92 | AL590842.1_CDS_1781 | 7 | 467 | 690 | 245 | 730 | 1.00E-127 | 46.26 |
| *Y. pestis* KIM10+ | AE009952.1_CDS_2457 | 7 | 467 | 690 | 245 | 730 | 1.00E-127 | 46.26 |
| *Y. pseudotuberculosis* IP31758 | CP000720.1_CDS_2233 | 1 | 746 | 969 | 1 | 730 | 0 | 51.28 |
| *Y. pseudotuberculosis* IP32953 | BX936398.1_CDS_1693 | 1 | 746 | 969 | 1 | 730 | 0 | 51.41 |
| *Y. rohdei* YRA | CP009787.1_CDS_1058 | 162 | 736 | 1086 | 55 | 640 | 1.00E-126 | 39.07 |
| *Y. rohdei* YRA | CP009787.1_CDS_1742 | 139 | 624 | 822 | 54 | 544 | 1.00E-152 | 49.3 |
| *Y. similis* Y228 | CP007230.1_CDS_3220 | 1 | 751 | 974 | 1 | 730 | 0 | 51.28 |

Supplementary Table 6: List of spacers found in *Yersinia* species that are similar to known plasmid sequence in *Yersinia*, together with their respective BLAST output.

| Genome | Spacer start position | Spacer end position | Percentage of identity (%) | Query start position | Query end position | Subject start position | Subject end position | Subject accession number and description |
| --- | --- | --- | --- | --- | --- | --- | --- | --- |
| *Y. frederiksenii* Y225 | 1745612 | 1745642 | 95 | 11 | 30 | 46580 | 46561 | gi|169544164|ref|NC_010377.1|  *Y. enterocolitica* plasmid pYE854 |
| *Y. frederiksenii* Y225 | 1756277 | 1756308 | 96.88 | 1 | 32 | 3644 | 3675 | gi|169544164|ref|NC_010377.1|  *Y. enterocolitica* plasmid pYE854 |
| *Y. frederiksenii* Y225 | 1756277 | 1756308 | 96.88 | 1 | 32 | 8819 | 8850 | gi|169544164|ref|NC_010377.1|  *Y. enterocolitica* plasmid pYE854 |
| *Y. frederiksenii* Y225 | 1756277 | 1756308 | 96.88 | 1 | 32 | 10785 | 10816 | gi|169544164|ref|NC_010377.1|  *Y. enterocolitica* plasmid pYE854 |
| *Y. frederiksenii* Y225 | 1756277 | 1756308 | 96.88 | 1 | 32 | 12751 | 12782 | gi|169544164|ref|NC_010377.1|  *Y. enterocolitica* plasmid pYE854 |
| *Y. kristensenii* Y231 | 26745 | 26776 | 96.88 | 1 | 32 | 3675 | 3644 | gi|169544164|ref|NC_010377.1|  *Y. enterocolitica* plasmid pYE854 |
| *Y. kristensenii* Y231 | 26745 | 26776 | 96.88 | 1 | 32 | 8850 | 8819 | gi|169544164|ref|NC_010377.1|  *Y. enterocolitica* plasmid pYE854 |
| *Y. kristensenii* Y231 | 26745 | 26776 | 96.88 | 1 | 32 | 10816 | 10785 | gi|169544164|ref|NC_010377.1|  *Y. enterocolitica* plasmid pYE854 |
| *Y. kristensenii* Y231 | 26745 | 26776 | 96.88 | 1 | 32 | 12782 | 12751 | gi|169544164|ref|NC_010377.1|  *Y. enterocolitica* plasmid pYE854 |
| *Y. kristensenii* Y231 | 37411 | 37442 | 95 | 3 | 22 | 46561 | 46580 | gi|169544164|ref|NC_010377.1|  *Y. enterocolitica* plasmid pYE854 |
| *Y. similis* Y228 | 4570301 | 4570332 | 100 | 16 | 30 | 47798 | 47784 | gi|386307331|ref|NC_017565.1|  *Y. enterocolitica* Y11 plasmid pYVO3 |
| *Y. similis* Y228 | 4570301 | 4570332 | 100 | 16 | 30 | 51854 | 51868 | gi|113911685|ref|NC_006153.2|  *Y. pseudotuberculosis* IP32953 pYV plasmid |
| *Y. similis* Y228 | 4570301 | 4570332 | 100 | 16 | 30 | 24934 | 24948 | gi|32470270|ref|NC_005017.1|  *Y. enterocolitica* 8081 plasmid pYVe8081 |
| *Y. similis* Y228 | 4570301 | 4570332 | 100 | 16 | 30 | 34597 | 34611 | gi|16082691|ref|NC_003131.1|  *Y. pestis* CO92 plasmid pCD1 |
| *Y. pseudotuberculosis* IP32953 | 2964877 | 2964909 | 91.3 | 1 | 23 | 2999 | 2977 | gi|410687978|ref|NC_019234.1|  *Y. enterocolitica* (type O:8) plasmid pYV-WA314 |
| *Y. pseudotuberculosis* IP32953 | 2964877 | 2964909 | 91.3 | 1 | 23 | 9183 | 9161 | gi|386307331|ref|NC_017565.1|  *Y. enterocolitica* Y11 plasmid pYVO3 |
| *Y. pestis* CO92 | 1773743 | 1773774 | 100 | 2 | 16 | 10555 | 10541 | gi|386307331|ref|NC_017565.1|  *Y. enterocolitica* Y11 plasmid pYVO3 |
| *Y. pestis* CO92 | 1773743 | 1773774 | 100 | 2 | 16 | 2836 | 2822 | gi|122815790|ref|NC_008791.1|  *Y. enterocolitica* 8081 plasmid pYVe8081 |
| *Y. pestis* KIM10+ | 2875809 | 2875840 | 100 | 17 | 31 | 10541 | 10555 | gi|386307331|ref|NC_017565.1|  *Y. enterocolitica* Y11 plasmid pYVO3 |
| *Y. pestis* KIM10+ | 2875809 | 2875840 | 100 | 17 | 31 | 2822 | 2836 | gi|122815790|ref|NC_008791.1|  *Y. enterocolitica* 8081 plasmid pYVe8081 |

Supplementary Table 7: Average nucleotide identity (ANI) value between pYV plasmids from different pathogenic *Yersinia* species.

|  | Y. pestis CO92 | Y. pseudotuberculosis  IP 32953 | Y. enterocolitica 8081 | Y. enterocolitica Y11 |
| --- | --- | --- | --- | --- |
| Y. pestis CO92 | --- | 99.33 | 97.58 | 96.96 |
| Y. pseudotuberculosis IP 32953 | 99.49 | --- | 97.21 | 96.62 |
| Y. enterocolitica 8081 | 97.65 | 97.54 | --- | 98.2 |
| Y. enterocolitica Y11 | 97.5 | 97.38 | 98.2 | --- |

Supplementary Table 8: GC and GC3 (third based in each codon) percentage of pYV plasmid.

| Strain name | GC% | GC3% |
| --- | --- | --- |
| Y. pestis CO92 | 45.65 | 45.04 |
| Y. pseudotuberculosis IP 32953 | 45.47 | 44.64 |
| Y. enterocolitica 8081 | 45.65 | 44.46 |
| Y. enterocolitica Y11 | 44.38 | 42.49 |
